# The prognostic significance of tertiary lymphoid structures in oral squamous cell carcinomas: a systematic review

**DOI:** 10.3389/froh.2024.1524313

**Published:** 2025-01-22

**Authors:** V. Ribeiro, J-L. Teillaud, M-C. Dieu-Nosjean, G. Lescaille, J. Rochefort

**Affiliations:** ^1^ Faculté de Santé, UFR Odontologie, Université Paris-Cité, Paris, France; ^2^ UMRS 1135, Faculté de Santé Sorbonne Université, Sorbonne Université, Paris, France; ^3^ INSERM Unit 1135, Paris, France; ^4^ Laboratory “Immune Microenvironment and Immunotherapy”, Centre D’Immunologie et des Maladies Infectieuses-Paris (CIMI-Paris), Paris, France; ^5^ Service Odontologie, Assistance Publique Hôpitaux de Paris (AP-HP), Hôpital Pitié-Salpêtrière, Paris, France

**Keywords:** oral cancer, oral oncoimmunology, oral squamous cell carcinoma, prognostic biomarker, tertiary lymphoid structures

## Abstract

**Introduction:**

Upper aerodigestive tract cancers are prevalent, with a global incidence surpassing 500,000 new cases in 2018. Among these, oral squamous cell carcinomas (OSCC) constitute the majority. OSCC has a low 5-year survival rate due to late-stage diagnosis. Risk factors include alcohol and tobacco use. However, non-smokers and non-drinkers are also affected, especially young patients with tongue cancer. The impact of tumor microenvironment (TME) and tumor-infiltrating lymphocytes (TILs) on OSCC prognosis remains debated. Remarkably, Tertiary Lymphoid Structures (TLS) identified in solid tumors have shown associations with favorable outcomes, yet their prognostic significance in OSCC remains understudied.

**Objective:**

Thus, this systematic review aims to explore the value of TLS in OSCC reported in the literature.

**Method:**

A scoping review was conducted and six retrospective cohort studies involving 1,203 patients met the inclusion criteria.

**Results:**

Predominantly male patients, with an average age of 49.3 years were included. Immunohistochemistry was the primary method to identify TLS, present in 21% up to 100% of cases. TLS were predominantly located in the peri-tumoral area (75.4%–84.8%) compared to the intra-tumoral area (33.8%–33.9%). Our review shows that the presence of TLS is associated with improved survival in OSCC.

**Discussion:**

However, variations in TLS detection and classification methods across studies introduce potential biases, hindering direct comparisons between findings. For instance, reports that are based solely on examining HES-stained slides for TLS identification may raise reliability concerns. Standardization of methodologies is imperative to ensure consistency in criteria utilization, thereby facilitating meaningful data comparisons.

**Systematic Review Registration:**

https://www.crd.york.ac.uk/prospero/display_record.php?ID=CRD42023428010, PROSPERO (CRD42023428010).

## 1 Introduction

The incidence of upper aerodigestive tract cancers in 2018 was over 500,000 new cases per year in the world (1–4). They are the 18th most common cancer in the world (2) and the 6th in France and are two to three times more common in men than in women (5). More than 90% of these cancers are squamous cell carcinomas grouping different locations (mainly the oropharynx and oral cavity, then the cavum and larynx). In a quarter of the cases, they are oral squamous cell carcinomas (OSCC). Mortality is estimated at 170,000 patients/year (6). OSCC has a low 5-year survival rate, below 50%, mainly due to late diagnosis (stages III/IV), which makes management difficult. Survival also depends on gender (better in women than in men) and tumor location (higher for lip cancer and lower for tongue cancer) (7). The main risk factors for OSCC are alcohol and tobacco consumption, and smokeless tobacco too (8), especially when combined (9). However, these cancers can also occur in patients who do not have these risk factors, particularly in young patients under 45 years of age (10, 11), and especially in tongue cancers in young women (12). These patients, with no “classic” risk factors (non-drinkers and non-smokers), present epidemiological and clinical characteristics distinct from smoker-drinker patients (10). The causes of cancer are not identified in these patients. Strikingly, the incidence of cases in young patients has been increasing in the last decade. Unlike oropharyngeal carcinomas (13, 14), human papillomavirus (HPV) is not a risk factor for OSCC.

Since the early 2000's, the management of cancer patients has been considering the immune tumor microenvironment (TME) as well as the immune response in addition to tumor-specific parameters (size and extension). TME varies from patient to patient and is now considered as a key parameter for cancer prognosis and patient response to immunotherapy. In various solid cancers, in particular in colorectal cancer, an “immunoscore” of immune cells present in TME is considered to have a prognostic value superior to that provided by the TNM classification (15–17). Studies of the OSCC TME have indicated that the influence of tumor-infiltrating lymphocytes (TILs), including regulatory T cells (Treg) and, for non-smoker and non-drinker patients, CD8^+^ T lymphocytes, is linked with improved overall survival (OS) (18). By contrast, It has been shown that a significant accumulation of peripheral IL-17^+^ T lymphocytes and higher IL-17 production in patients with head and neck cancer are negatively correlated with OS (19). A higher proportion of circulating T helper 17 (Th17) lymphocytes is found in patients with OSCC, with an increase in Th17/Tregs ratio in early stages and a decrease in this ratio in higher stages of oral cancer (20).

When the architecture and organization of TME in solid tumors were examined, the presence of tertiary lymphoid structures (TLS) was first evidenced and associated with more favorable prognosis in non-small cell lung carcinoma (NSCLC) (21). TLS are ectopic and transitory structures appearing within non-lymphoid tissues in a context of inflammation, especially when the latter is chronic. The term “tertiary lymphoid tissue” was first used in 1992 by Picker and Butcher, who described the formation of lymphocyte aggregates outside of lymphoid tissue, bringing together memory lymphocytes and/or their precursors (22). In 1996, Schröder and her colleagues described B cell differentiation in ectopic lymphoid structures organized in the same way as lymph nodes, possibly mimicking their function in rheumatoid arthritis (23). The same year, Ruddle and her colleagues showed in a murine model that chronic inflammatory lesions caused by lymphotoxin resembled lymph nodes with regard to cellular composition, T and B cell zones, primary and secondary follicles, presence of high endothelial venules, and ability to respond to antigenic challenge (24). Many reports have now shown that tumor-associated TLS participate in the mounting of adaptive immune responses resulting in the differentiation of cytotoxic effector T cells and the production of anti-tumor antibodies (25). The non-encapsulation of TLS, unlike lymph nodes, is likely to allow for easier migration of immune cells, resulting in improved antigens addressing and the accumulation of new antigen specificities, which in turn promotes the persistence of these structures (26).

A recent bibliographic study on global trends in TLS research highlighted the growing interest in these structures, with a significant increase in publications between 2017 and 2023. The main contributing countries were China (231 publications), the United States of America (212 publications), and France (89 publications). The most productive institutions were French, including Inserm, followed by Université Paris Cité and Sorbonne Université. Key topics encompassed the links between TLS and cancers, immunotherapy, the tumor microenvironment, prognosis, and the responsiveness to immune checkpoint inhibitors (27).

TLS presence is mostly synonymous with a better survival prognosis in various solid tumors such as NSCLC, melanoma, breast cancer, and many digestive cancers (21, 25, 28), except for a rare subtype of hepatocellular carcinoma (29), although the latter has not been confirmed by other studies so far. Remarkably, it has been demonstrated that CD8^+^ lymphocytes are associated with more favorable prognosis only when TLS are present, in NSCLC (30) and ovarian cancers (31). Furthermore, better responses to immunotherapy have been observed when stimulating TLS formation (32) or when TLS are present in cancer patients receiving anti-immune checkpoint antibodies (anti-PD1, anti-PD-L1, and anti-CTLA-4) (33–35).

Studies have also focused on the regulation of TLS by Tregs. Using a murine model, Joshi and his colleagues have reported that the formation of TLS can be inhibited by Tregs (36). It has been also recently shown that Tregs infiltrate TLS in a number of NSCLC patients and are then associated with a poor clinical outcome (37).

However, to our knowledge, no published study has comprehensively synthesized data regarding the protective role of TLS in OSCC. To gain deeper insights into the potential significance of TLS as a prognostic marker in OSCC, we undertook this systematic review to compile all available data on the subject. The primary aim of this review was to investigate the potential link between TLS presence and OSCC prognosis. Additionally, the secondary objective was to evaluate whether the organization and/or tissue localization of TLS influence the prognosis of OSCC patients.

## 2 Materiel and methods

A systematic review was conducted between January 2022 and May 2023. The research protocol was registered on the International Prospective Register Of Systematic Reviews PROSPERO on number CRD42023428010.

### 2.1 Search strategy

This is a scoping review of the literature according to the Joanna Briggs Institute guide (38). The methodology of the Scoping Review met PRISMA requirements (39). We have used the following databases: Pubmed, Embase, Scopus, and Web of Science. The same research was performed on the grey literature (OpenGrey and Google Scholar). The complete search strategy is available in Supplementary Appendix 1. No time restriction was selected.

#### 2.1.1 Complementary research

An additional search was conducted by replacing the OSSC keywords with head and neck squamous cell carcinoma (HNSCC) keywords to find articles that mainly focus on OSCC patients. Two authors of the present study independently performed data extraction, with a third author consulted in case of disagreement. However, it is important to note that HNSCC included both OSCC and oropharyngeal cancers, which have distinct immunological characteristics (40). Therefore, we only included articles that provided detailed data specifically related to OSCC or where OSCC comprised the majority of HNSCC cases (more than 75%).

### 2.2 Definition of variables

OSCC refers to cancers of the oral cavity derived from the epithelial cells of the oral mucosa. These epithelial neoplasms exhibit varying degrees of differentiation and tend to local and regional invasion. Typically, squamous carcinoma cells are detected by a pan-cytokeratin marker (41). As stressed in the Introduction section, TLS are ectopic and transitory structures that appear within non-lymphoid tissues in the context of inflammation, particularly in cases of chronic inflammation. Histologically, TLS consist of clusters of B and T lymphocytes with dendritic cells, organized as in secondary lymphoid organs (24). Their organization can vary widely, from loosely aggregated lymphocytes to well-formed structures featuring a T-cell and a B-cell zones. Within the T-cell zone, diverse T-cell subsets can be detected, marked by the presence of follicular helper T-cells (Tfh) (42), alongside with mature dendritic cells (mDC). The B-cell zone includes a network of follicular dendritic cells (FDC) and a germinal center where class switch recombination (CSR) and hypersomatic mutations (HSM) occur. This organizational framework is complemented by high endothelial venules (HEV), supplying the necessary cellular components and chemokines essential for the formation of mature TLS (25).

### 2.3 Inclusion criteria

To be selected, the articles needed to focus on studying the presence of TLS in human OSCC samples as the primary outcome and evaluate the difference in prognosis between samples with and without TLS (classified by “yes” or “not” or “high” or “low” or “classic” and “non-classic” or “mature” or “immature”) and using survival index *like overall survival* (OS). Studies were eligible for inclusion with no period restriction. We also made no language restrictions when an English translation was available. We therefore included experimental and observational studies.

### 2.4 Exclusion criteria

We excluded review articles, editorial notes, and studies involving only animal or *in vitro* models from our selection.

### 2.5 Data extraction

The titles and abstracts were first selected by screening various databases (see « Search Strategy ») to extract articles that respected the inclusion criteria. A full-text reading of these articles was then performed to select those to be included in the systematic review. Full-texts of articles with a degree of uncertainty with regard to inclusion criteria were also read. The references listed in the selected articles were then analyzed to include additional papers not identified by our initial search. Details of each study, design, participant characteristics, intervention and comparator, and outcomes were also extracted. Softwares used to perform this reference-based search were EndNote (version 21.4, London, United Kingdom) and Zotero (version 7.0.8, Virginia, United States of America (USA)) for ranking and referencing articles, and Rayyan (version 2023, Cambridge, Massachusetts, USA) for the screening.

#### 2.5.1 Quality assessment

We have assessed the levels of evidence of the articles obtained by applying the Haute Autorité de Santé (HAS) literature analysis guide (43), which categorizes each article according to its rigor and reliability as providing low, intermediate, or high levels of proof. This guide helps to standardize the evaluation of scientific literature, ensuring that conclusions drawn are based on evidence of varying strengths. Articles with a high level of proof typically have more rigorous methodologies and stronger outcomes, while those with lower levels may have limitations or less robust methodologies. By grading each article, authors can better interpret the reliability and validity of the collective findings, making informed decisions based on the weight of the evidence provided.

## 3 Results

### 3.1 Literature search description and main characteristics of the cohorts

**Figure 1:**
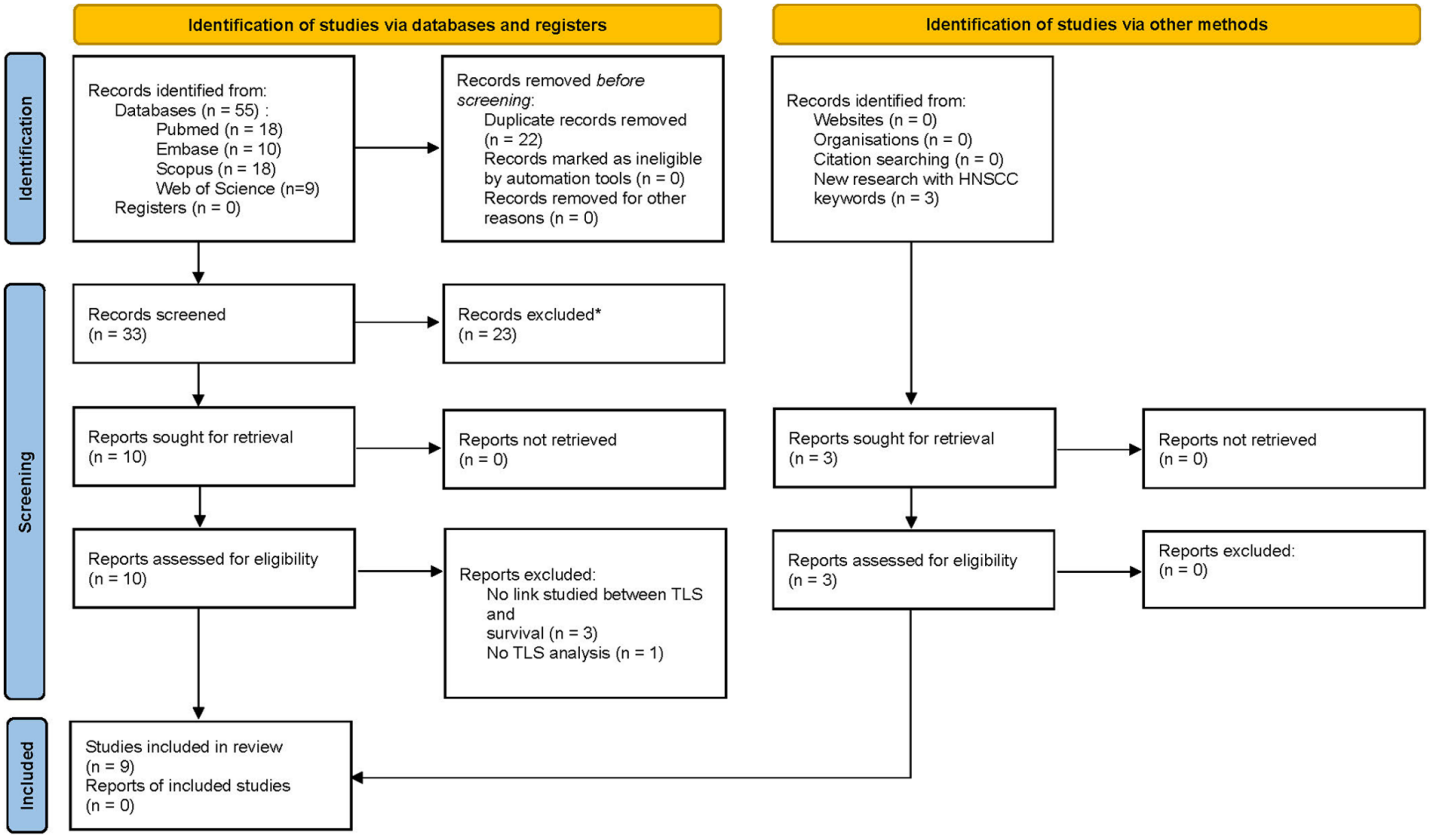
PRISMA 2020 flow for new systematic reviews which included searches of databases, registers and other sources.

After completing the literature search using various databases and relevant keywords, fifty-five publications were identified: we obtained eighteen articles from the PubMed database and, using the same keywords, also eighteen from the Scopus transdisciplinary database, ten from EMBASE, and nine from the Web of Science. The Cochrane database did not allow us to include any articles on the topic. Finally, after removing duplicates, thirty-three articles were selected (Figure 1). Of these 33 papers, only ten were eligible for abstract screening based on title selection. The others did not meet our inclusion criteria as *their titles and/or abstracts did not mention a prognostic study related to the presence of TLS.* All the ten articles were eligible for full-text screening. It led to the selection of six articles (44–49), the four others being excluded for different reasons (some studies did not include a survival analysis related to the presence of TLS, and others did not include a study of TLS). Three other articles (50–52) were added as the result of the complementary research as indicated in Figure 1.

*Thus, the bibliographic search identified nine articles* (44–52) *focusing on the study of TLS presence in human OSCC samples and evaluating the prognostic differences between samples with and without TLS*. Eight of them were retrospective cohort studies and one was a prospective cohort study. These nine articles presented an intermediate level of proof*, with a grade of 4C, except for one study, which was graded 2B,* according to the HAS (43) which means that, although the studies have a certain reliability and provide relevant results, they present an acceptable but not optimal level of rigor, which may imply methodological limitations. Nevertheless, they provided a valuable contribution to our overall conclusions.

### 3.2 Clinicopathological characteristics of the OSCC patients and types of tumors

Our review included a total of 1,203 patients, with each study having a cohort size ranging from *n* = 9 to *n* = 310 individuals. Among them, 745 (61.93%) patients were male and 458 (38.07%) were female. The mean age was 49.3 years (ranging from 23–83 years). The other clinicopathological and demographic data of the patients are presented in Table 1. Most of the samples analyzed consisted of early-stage tongue OSCCs, with proportions ranging from 32.98%–85.85% (Table 1). In one study, 11 out of 65 samples were not OSCC, comprising four adenocarcinomas, four classified as “other,” and three unknown cases (45). Regarding tumor location, some studies focused specifically on tongue OSCCs (47, 49), whereas others examined tumors involving the lips, gums, floor of the mouth, palate, or oral mucosa.

**Table 1:** Clinico-pathological and demographic data of patients.

Author/Year of publication/Country	Number of patients	Sex Ratio (M/F)	Mean age (min-max) in years	Tumor site (number and proportion of patients)	Risk factors	
					Tobacco	Alcohol
Wirsing et al. Norway (44)	80	46/34	63	Tongue: 38 (47.5%)	Never: 18	Never: 13
				Buccal mucosa: 8 (10%)		
				Gingiva: 10 (12.5%)	Former: 11	≤1/week: 30
				Oral floor: 22 (27.5%)	Current: 45	>1/week: 20
				Others: 2 (2.5%)	Unknown: 6	Unknown: 17
Li et al. China (45)	65	44/21	NR	Tongue: 23	NR	NR
				Buccal mucosa: 13 (20%)		
				Oral Floor: 8 (12.3%)		
				Gingiva: 14 (21.5%)		
				Others: 7 (10,8%)		
Li et al. China (46)	168	120/48	57 (24–83)	Tongue: 73 (43,5%)	Never/Former: 84	Never/Former: 91
				Buccal: 39 (23,2%)		
				Gingiva: 37 (22%)		
				Others: 19 (11.3%)	Current: 84	Current: 77
Wang et al. China (47)	97	46/51	53 (23–79)	Tongue: 97 (100%)	NR	NR
Peng et al. China (48)	106	67/39	56 (25–79)	Tongue: 91 (85.9%)	No: 60	NR
				Buccal: 3 (3%)		
				Gingiva: 7 (7%)		
				Oral floor: 4 (3.7%)	Yes: 46	
				Others: 1 (1%)		
Almangush et al. Finland (49)	310	164/146	62	Tongue: 310 (100%)	NR	NR
Wang et al. China (50)	188	111/77	NR	Tongue: 62 (33%)	NR	NR
				Buccal: 53 (28.2%)		
				Gingiva: 29 (15.4%)		
				Oral floor: 11 (5.9%)		
				Others: 19 (11.3%)		
Weed et al. USA (51)	9	7/2	71	Tongue: 4 (44.4%)	No: 4	NR
				Buccal: 2 (22.2%)		
				Gingiva: 1 (14.4%)		
				Oral floor: 11 (5.9%)	Yes: 5	
				Others: 2 (22.2%)		
Li H. et al. China (52)	180	140/40	60	Tongue: 87 (48.3%)	No: 70	No: 92
				Buccal: 34 (18.9%)		
				Gingiva: 26 (14.4%)		
				Oral floor: 18 (10%)	Yes: 110	Yes: 88
				Others: 5 (2.8%)		

NR, Not Reported.

### 3.3 Identification of TLS and quantification

The presence of TLS was reported in 21.0%–100% of cases across studies, considering both intra-tumoral and peri-tumoral locations (Table 2). The methods used to identify TLS varied significantly among the nine studies analyzed, as summarized in Table 3. One study exclusively employed hematoxylin-eosin-safranin (HES) staining to detect and quantify TLS, whereas the others used immunohistochemical (IHC) labeling to confirm their presence (49). Common markers utilized included CD20 for B cells, CD3 for T cells, CD21 to highlight follicular dendritic cell (FDC) networks, DC-Lamp to detect mature dendritic cells, and PNAd for high endothelial venules (HEVs). Some studies also employed the Bcl-6 marker to confirm the presence of germinal centers (44, 47). For studies using HES staining, evaluations were performed by trained pathologists or researchers (46, 47, 49). However, one study described only the presence of TLS within (intra-tumoral) or outside (peri-tumoral) tumor region, which limited the ability to evaluate the overall prevalence of these structures. This study found intra-tumoral TLS in 33.8% of cases compared to 75.4% for peri-tumoral TLS (45).

**Table 2:** Results of the studies included in the systematic review.

Publication	Study design	Presence of TLS (%)	Main results
Wirsing et al. (44)	RC	21%	TLS associated with a better OS (*p* = 0.039)
Li et al. (45)	RC	33.8% in ITA 75.4% in PTA	TLS associated with a better OS (*p* = 0.006) and DFS (*p* = 0.009)
Li et al. (46)	RC	26.8%	TLS associated with a better OS (*p* < 0.001) and DFS (*p* = 0.002),
Wang et al. (47)	RC	73.6%	TLS presence is an independent prognostic factor for OS (HR = 0.434, 95% CI = 0.212–0.886, *p* = 0.022) and DFS (HR = 0.459, 95%CI = 0.235–0.897, *p* = 0.023)
Peng et al. (48)	RC	32.08%	TLS associated with a better OS and PFS (*p* < 0.05).
Almangush et al. (49)	RC	33.9% in ITA 84.8% in PTA	Peritumoral TLS associated with better DSS (HR 1.96, 95% CI 1.09–3.54; *p* = 0.025) and OS (HR 1.66, 95% CI 1.11–2.48; *p* = 0.014).
Wang et al. (50)	RC	56.91%	TLS associated with better OS (*p* < 0.001) and DFS (*p* = 0.019) that was improved when TLS maturity is taken in account.
	TCGA	100%	TLS score: protective factor for OS [HR = 0.142 (95% CI 0.024–0.858), *p* = 0.033].
Weed et al. (51)	PC	100%	Plasma cells dominate TLS1 interactions, which are significantly correlated with a better recurrence-free survival.
Li H. et al. (52)	RC	39.45%	Patients with TLS^+^ TME associated with a better OS than those with TLS^–^ TME (*p* = 0.0058)
	TCGA	NR	Patients with immunotype of TME enriched with TLS signature: better prognosis

DFS, disease-free survival; HES, hematoxyline-eosine-safran; IHC, immunohistochemistry; ITA, intratumoral area; LTB, lymphotoxin beta; mIF, multiplexed immuno-fluorescence; OS, overall survival; OTSCC, oral tongue squamous cell carcinomas; PC, prospective cohort; PFS, progression-free survival; PTA, peritumoral area; RC, retrospective cohort; RT-PCR, reverse-transcriptase-polymerase chain reaction; TCGA, the cancer genome Atlas; TIL, tumor infiltrating lymphocyte; TME, tumor microEnvironment; TLS, tertiary lymphoid structure. *China: People's Republic of China (PRC).

**Table 3:** Method of identification, labeling, and ranking of TLS (Tertiary Lymphoid Structures) among the different studies.

Publication	Technique and applied markers		Method of TLS classification	
			Based on TLS maturity	Based on TLS location
Wirsing et al. (44)	IHC	• CD20, CD-3 (B/T Ly),	• No B cells agg. = TLS (−)	Peritumoral vs. Intratumoral
		• CD21 (FDC),	• Aggregates of B cells +/− t GC:	
		• DC-Lamp (mDC),	- CD21(+/−) = non-classic TLS	
		• PNAd (HEV) Bcl-6 (GC-B cells)	- CD21(++) = classic TLS	
Li K. et al. (45)	HES	• CD20, CD3 (B/T Ly),	No maturity classification	• 0: No TLS
				• 1: P-TLS (1–4) + no I-TLS.
	IHC	• CD21 (FDC),		• 2: P-TLS > 4 +no I-TLS.
				• 3: I-TLS (1–4)
				• 4: I-TLS > 4.
Li Q. et al. (46)	IHC	• CD20, CD3 (B/T Ly),	0 = No follicle	No spatial classification
		• DC-Lamp (mDC),	1 = Scattered CD20^+^/CD3^+^ = Immature TLS	
	mIF	• PNAd (HEV)	2 = Follicle CD20^+^ = Mature TLS	
Wang et al. (47)	HES	• CD20, CD-3 (B/T Ly),	• E-TLS: dense lymph. Agg.- no FDCs	No spatial classification
		• CD21 (FDC),	• PFL-TLS: B cells + FDC + no GCs	
	IHC	• Bcl-6 (GC)	• SFL-TLS, B cells + FDC and GCs	
		• Bcl-2 (prolif. marker)	• No TLS E-TLS = Low-Mat. gr	
			PFL-TLS + SFL-TLS = High-Mat. gr	
2021 Peng et al. (48)	HES	• CD20 (B cells)	No maturity or spatial classification	
	IHC			
	mIF	TCF7^+^ cell interaction	NC	NC
Almangush et al. (49)	HES	NC	1 = No TLS	NC
			2 = Lymph. agg./s: ill-defined clusters of Ly	
			3 = Primary follicle (without GC)	
			4 = Secondary follicle	
Wang et al. (50)	HES	• CD20, CD3 (B/T Ly),	On HES:	Peritumoral vs. Intratumoral
			• TLS^−^: No TLS	
			• Immature TLS^+^: Lymphocytes gathered without clear boundaries	
			• Mature TLS^+^: Presence of GC	
			On IHC:	
	IHC	• PNAd (HEV)	• Grade 1 TLS: T cells in the center and B cells agg. or scattered around the T-cell zone	
			• Grade 2: B cells and T cells gathered into clusters - boundary between them unclear	
			• Grade 3 TLS: B cells into the center formed follicular structures with T cells surrounding.	
Weed et al. (51)	CODEX	• CD3 (T Ly)	• TLS1: plasma cells and CD4^+^FoxP3^−^ Ly.	No spatial classification
		• CD4 (Th Ly)		
		• CD8 (Tc Ly)	• TLS2: enriched in M2 and Treg	
Li et al. (52)	mIF	• CD20, CD3 (B/T Ly),	No maturity or spatial classification	
		• DC-Lamp (mDC)		
		• TCF1 (exhausted T Ly)		

E-TLS, early-tertiary lymphoid structure, FDC: follicular dendritic cell, GC: germinal center, lymph.: lymphocytic, agg.: aggregates, HES: hematoxyline-eosine-safran, HEV, high endothelial venule, IHC: immunohistochemistry, Prolif.: proliferation, mat. gr: maturity group, mIF: multiplexed immuno-fluorescence, NC: not concerned, PFL-TLS: primary follicle like-tertiary lymphoid structure, PNAd: peripheral node addressin, RT-PCR: reverse-transcriptase-polymerase chain reaction, SFL-TLS: secondary follicle-like tertiary lymphoid structure, TLS: tertiary lymphoid structure, Tc Ly: T-cytotoxic lymphocyte, Th Ly: T-helper lymphocyte, Treg: T regulatory lymphocytes, P-TLS: peritumoral TLS, I-TLS: intratumoral TLS, Ly: lymphocyte, M2: macrophages. *China: People's Republic of China.

Classification of TLS and their degree of maturity also varied considerably between studies. Some classifications were based on structural organization, while others relied on the location of TLS relative to the tumor or their functional maturity. Certain works distinguished between mature TLS, characterized by a well-organized structure with a B-cell follicle containing a germinal center surrounded by a T-cell zone, and immature TLS, which consisted of less organized aggregates of lymphocytes and dendritic cells (44, 46). Other studies refined this classification by introducing three levels of maturity: dense lymphocyte aggregates without FDCs, TLS with a germinal center, and TLS containing a B-cell follicle and dense FDC network. The frequency of these TLS types was inversely proportional to their degree of maturity, with immature structures being the most common (47). Another approach followed a similar methodology, initially detecting TLS via HES staining before classifying their organization based on IHC observations (49).

Some of the studies focused on the location of TLS as a key classification criterion. One study categorized TLS according to their presence within, adjacent to, or in both regions relative to the tumor. Tumors were then graded based on TLS density and distribution, with grades ranging from 0 (absence of TLS) to 4 (high density and complexity) (45). Another work, in contrast, did not consider TLS maturity but rather categorized tumors as TLS-positive or TLS-negative, based on IHC and immunofluorescence findings. TLS density was also quantified by normalizing their number to the tumor surface area, with TLS-positive tumors reported in 32.08% of cases (48). A different approach investigated the relationship between TLS and TME. Two distinct TME profiles associated with TLS were identified. The first was enriched in plasma cells and CD4^+^ FoxP3^−^ T lymphocytes, alongside monocytes, neutrophils, and M1 macrophages. The second was characterized by an abundance of M2 macrophages and Treg cells, accompanied by other immune cell populations, including CD4^+^ and CD8^+^ T cells, NK cells, and neutrophils (51).

Clearly, this variability in methodologies and classification systems underscores the need for standardization in TLS evaluation. Differences in detection techniques, markers, and definitions of maturity or location may significantly impact the interpretation of TLS prevalence, organization, and their functional relevance within tumor microenvironments.

### 3.4. Survival analysis

#### 3.4.1 Association between TLS presence and overall survival (OS)

The presence of TLS was shown to significantly correlate with better OS in several studies. One study demonstrated that patients with TLS had an 88.2% 5-year survival rate compared to 60.3% for TLS-negative patients. However, this association was statistically significant only when TLS were observed at multiple slice levels (*p* = 0.039), whereas a single slice level showed only a non-significant trend towards improved prognosis (44). Another study reported a significant association between TLS and long-term survival (*p* = 0.006), with survival curves showing longer OS in patients with more advanced TLS maturation stages (*p* = 0.031). The 5-year OS rates for patients without TLS, with peri-tumoral TLS, and with intra-tumoral TLS were 45.9 ± 12.4 months, 59.2 ± 3.9 months, and 76.1 ± 3.1 months, respectively (45). Finally, another analysis found a significant difference in the 5-year OS rates between TLS-positive and TLS-negative groups (88.9% vs. 56.1%; *p* < 0.001), confirmed by multivariate analysis (HR = 3.784, 95% CI, 1.498–9.562) (46).

#### 3.4.2 Impact of TLS maturity

The maturity of TLS also appears to play a role, although its influence varies across studies. “Classic” (mature) and “non-classic” (immature) TLS were both associated with better 5-year OS, but patients with mature TLS tended to have lower disease-specific mortality, though this was not statistically significant (*p* = 0.304) (44). In contrast, another study found no significant impact of TLS maturity on OS (94.1% vs. 85.7%, *p* = 0.411) (46). A further analysis showed a statistically significant difference in 5-year OS between patients with “low-maturity” TLS (63.4%) and those with “high-maturity” TLS (96%) (*p* = 0.0176) (47).

#### 3.4.3 Gene expression and immunological features

The association between TLS and better OS was also linked to molecular and immunological features. One study identified the overexpression of IL-7, CXCL-13, and lymphotoxin beta (LTβ) in patients with advanced TLS maturation stages, supporting their positive impact on prognosis (45). Another study highlighted the presence of TCF7^+^ T cells within TLS, associated with improved prognosis (48). Gene expression analyses in a large TCGA database cohort further confirmed TLS as a protective factor for OS (HR = 0.142, 95% CI 0.024–0.858, *p* = 0.033) (50).

#### 3.4.4 Multivariate analyses and independent prognostic value

Multiple studies confirmed the independent prognostic value of TLS. One analysis showed that TLS presence remained a protective factor for OS (HR = 0.434, 95% CI, 0.212–0.886, *p* = 0.022) in early-stage tongue OSCC (47). Another study reaffirmed that TLS positivity correlated with better OS (*p* < 0.001) and DFS (*p* = 0.019), with multivariate analysis identifying TLS score as an independent factor for OS (HR = 0.874, 95% CI, 0.765–0.997, *p* = 0.045) (50).

## 4 Discussion

Our work has highlighted several key points. Only a total of nine articles investigating the prognostic value of TLS in OSCC met our inclusion criteria. The reported presence of TLS ranged from 21.0%–100% of cases across the studies, with most research teams utilizing HES or IF for their detection. In all included studies, TLS were consistently associated with improved survival outcomes. We specifically focused on OSCC due to the scarcity of reviews addressing this topic and because OSCC represent the majority of cancers within the oral cavity (53). Within the broader category of HNSCC, there is considerable variability among cancer subtypes, including salivary gland cancers (54, 55) and oropharyngeal cancers, some of which are HPV-related (56–58). This diversity, combined with our emphasis on TLS, prompted us to narrow the scope of the study to OSCC. By doing so, we were able to concentrate on survival outcomes, detection techniques, and TLS classifications in this specific context.

Most cohorts were dominated by tongue OSCC cases, aligning with literature trends showing an increasing proportion of tongue cancers in OSCC cohorts (59–62). Studies also differed significantly in terms of tumor staging, which impacts survival (63, 64). Some studies included mostly advanced-stage cancers (18, 59), while others focused on early-stage cancers (60), likely due to recruitment bias. TME composition, influenced by tobacco and alcohol exposure, may also differ by tumor stage (18). Since the included studies did not stratify patients based on risk factors, this adds complexity to evaluating the prognostic value of TLS in OSCC. In one study, tumor staging was determined based on histological grades rather than the TNM classification system (45). Additionally, 11 specimens were not confirmed as OSCC, including four adenocarcinomas, four cases of “other tumors,” and three unidentified tumors. Despite the lack of stratified data on survival and TLS presence by cancer type, the study was included as over 83% of the samples were OSCC. Similarly, other included articles reported non-OSCC tumor types in 2.78%–22.22% of cases but were included because their cohorts were predominantly including OSCC patients.

*HES staining was the most commonly used technique to detect TLS. Among five studies using HES, only two relied on qualified pathologists, while the other ones used trained investigators.* Two studies clearly defined TLS presence on HES sections, classifying samples with at least one ill-defined lymphocyte aggregate (which means that samples containing at least one ill-defined lymphocyte aggregate are classified as positive)*, while others did not specify histological criteria. Immunostaining methods, including immunohistochemistry (IHC) and multiplexed immunofluorescence (mIF), were also employed, with consistent marker use across studies.* These markers are part of a quintet found in several articles attempting to highlight the different cell populations found in TLS (30, 65–71). Similarly to Wirsing et al., some authors also used the Bcl-6 marker to highlight germinal centers and confirm the presence of an active B-cell follicle. This methodology appears to be the most accurate for detecting histologically the presence of TLS with an active germinal center, as it allows for the assessment of TLS maturity, as explained in further detail below.

When examining the detection methods employed in the literature, it becomes evident that HES staining is the most used approach, sometimes combined with validation through IHC labeling. Although HES staining cannot differentiate between distinct lymphocyte populations, it remains a simple, rapid, and improved technique (71, 72). Moreover, its accuracy is maintained, particularly when performed by a qualified pathologist or trained examiner. One study used CODEX, a complex imaging technique that better reflects cellular interactions within the TME. This technique makes it possible to highlight many markers on the same slide, enabling more precise analysis of cell neighborhoods to identify different histological profiles. *Some studies also analyzed immune signatures in the TCGA database to assess TLS presence and prognostic value* in OSCC and HNSCC (47, 52). Regardless of the method used, concerning OSCC, the results are consistent, TLS correlated with a better prognosis.

TLS *classification* also varied among the studies, *focusing* on location, maturity, and specific cell populations involved. Maturity-based classification often distinguished between immature and mature TLS, with some studies going further by subtyping TLS based on various levels of maturity. Studies examining spatial distribution found higher TLS proportions in peritumoral stroma or at invasion margins, which is consistent with recent reviews (73, 74). However, one study contradicts *this by associating* intra-tumoral TLS with better prognosis (46). These discrepancies may be attributed to methodological and classification *differences as well as patient cohort characteristics. The lack of standardized criteria complicates comparisons between studies, highlighting the need for unified classification systems to enable consistent evaluations across cancer types*.

In terms of survival, all the studies demonstrated a positive association between the presence of TLS and improved OS, as well as better Recurrence-Free Survival (RFS) or Progression-Free Survival (PFS). However, variations in survival indicators used among the studies make direct comparisons challenging. *For example, one study defined disease-specific survival (DSS) as the time between diagnosis and death due to OSCC or at the last follow-up* (49)*, while another used DFS, which was defined as the time from surgery to disease relapse or death due to disease* (47)*. Li* et al. (45) *also employed this survival index but defined it as the time from surgery to the onset of local or metastatic recurrence. Thus, standardizing survival metrics would greatly improve data comparability and reliability.* However, survival results overwhelmingly indicated that OSCC patients with organized local immune responses in TLS have better survival outcomes. This finding is consistent with data reported in the literature for other solid tumors, including breast cancer (42, 67, 68, 75, 76), non-small cell lung cancer (21, 30, 66, 77), melanoma (35, 78, 79), hepatocellular carcinoma (80), pancreatic cancer (81), sarcoma (34), clear cell renal cancer (82) and colorectal cancer (83–90). Similarly, to the identification methods, standardizing patient survival assessments would facilitate more accurate comparisons of the data. These nine studies exhibit significant limitations. Notably, not all of them *accounted for risk factors like tobacco and alcohol exposure, treatment history, or patient comorbidities. These gaps further complicate the analysis of TLS as a prognostic factor*.

Last, immune checkpoint inhibitors targeting PD-1 (programmed cell death protein 1), such as nivolumab and pembrolizumab, are currently utilized for OSCC treatment (91, 92). Additional PD-1/PD-L1 axis inhibitory antibodies are currently under clinical investigation, such as cemiplimab (NCT04398524), sintilimab (NCT05000892), or toripalimab (NCT04825938). However, the clinical effectiveness of these therapies remains relatively low, ranging from 20%–40% depending on the cancer type. The causes of resistance to immunomodulators remain largely unknown. Among various factors, mature TLS may forecast the efficacy of immune checkpoint inhibitors in solid tumors (65). In soft tissue sarcomas, an immune classification rooted in the analysis of the TME has enabled the classification of tumors into immune-desert, immune-rich, or highly vascularized immune classes. The most immune-rich class is distinguished by a high expression of a B lymphocyte signature and the presence of TLS, correlating with enhanced survival (93). Beyond mere identification and prognostication of patient response capabilities, it might be feasible to induce TLS, allowing patients without these structures to develop them and thus benefit from improved therapeutic responses. This concept has been explored in murine models (32, 94, 95). It could potentially represent the future of personalized anti-cancer therapies. This underscores the importance of studying these structures.

## 5 Conclusion

Overall, the results of the literature review suggest that the presence of TLS is associated with better survival outcomes in oral squamous cell carcinoma. The variability in classification criteria and survival indicators highlights the need for standardized approaches in future research. Further studies with larger sample sizes and standardized methodologies are warranted to validate these findings and explore the underlying mechanisms of TLS in oral cancer.

## Data Availability

The original contributions presented in the study are included in the article/Supplementary Material, further inquiries can be directed to the corresponding authors.
