# Supplementary material for: The prognostic significance of tertiary lymphoid structures in oral squamous cell carcinomas: a systematic review
Source: Front Oral Health. 2025 Jan 22;5:1524313. doi: 10.3389/froh.2024.1524313 (PMC11794802; doi:10.3389/froh.2024.1524313)
Supplement: Supplementary file 1 [file Datasheet1.docx]

Tertiary lymphoid structure has prognostic factor in OSCC: A systematic review.

PECOS:

| P | Patients with OSCC |
| --- | --- |
| E | Tertiary Lymphoid Structure |
| C | - |
| O | Survival (Overall Survival, Progression Free Survival, Disease Free Survival) |
| S | Retrospective study (Cohort study, Case Control study, Translational Study) |

Keywords:

#1: Oral Squamous Cell Carcinoma

- Mouth Neoplasm (Mesh term)
- Oral Squamous cell carcinoma
- OSCC
- Oral cancer

#2: Tertiary Lymphoid Structure

- Tertiary Lymphoid Structure (Mesh Term)
- TLS
- Lymphoid aggregates
- Lymphoid organs
- Ectopic Lymphoid aggregate/organ/structure
- B-Cell Cluster/follicle
- tumor-associated immune aggregates

#3: Survival/Prognostic

- Survival (Mesh term)
- Prognostic (Mesh term)
- DFS: Disease free survival
- PFS: Progression Free Survival
- OS: Overall survival
- Prognostic

Equation de recherche

#1: Oral Squamous Cell Carcinoma

“Mouth Neoplasm” [Mesh Terms] OR “Mouth Neoplasm” OR OSCC OR “Oral Squamous Cell Carcinoma” OR “Oral Squamous Cell Carcinomas” OR “Squamous cell Carcinoma of the Tongue” OR “Squamous cell Carcinomas of the Tongue” OR “Oral Cancer” OR “Oral Cancers” OR “Oral Tongue Squamous Cell Carcinoma” OR “Oral Tongue Squamous Cell Carcinomas” OR “Oral Cavity Squamous Cell Carcinoma” OR “Oral Cavity Squamous Cell Carcinomas” OR “Squamous Cell Carcinoma of the Mouth” OR “Squamous Cell Carcinomas of the Mouth”

#2: Tertiary Lymphoid Structure

"Tertiary Lymphoid Structures"[Mesh] OR “Tertiary Lymphoid Structure” OR “Tertiary Lymphoid Structures” OR “Tertiary Lymphoid Organ” OR “Tertiary Lymphoid Organs” OR “Ectopic Lymphoid Follicle” OR “Ectopic Lymphoid Follicles” OR “Ectopic Lymphoid Organ” OR “Ectopic Lymphoid Organs” OR “Intratumoral Lymphoid Structure” OR “Intratumoral Lymphoid Structures” OR “Intratumoral Lymphoid Organ” OR “Intratumoral Lymphoid Organs” OR “Intratumoral Lymphoid Follicles” OR “Intratumoral Lymphoid Follicle” OR “Intra-tumoral Lymphoid Structure” OR “Intra-tumoral Lymphoid Structures” OR “Intra-tumoral Lymphoid Organ” OR “Intra-tumoral Lymphoid Organs” OR “Intra-tumoral Lymphoid Follicles” OR “Intra-tumoral Lymphoid Follicle” OR “B-Cells Follicle” OR “B-cells Follicles” OR “B-Cells Cluster” OR “B-Cell Cluster” OR “B-Cell Clusters” OR “B-Cells Clusters” OR “BCells Follicle” OR “Bcells Follicles” OR “BCells Cluster” OR “BCell Cluster” OR “BCell Clusters” OR “BCells Clusters” OR “tumor-associated immune aggregates” OR “tumor associated immune aggregates”

#3: Survival/ Prognosis

"Survival"[Mesh] OR "Disease-Free Survival"[Mesh] OR "Survival Analysis"[Mesh] OR "Survival Rate"[Mesh] OR "Progression-Free Survival"[Mesh] OR “Survival” OR “Survival Analysis” OR “Survival Rate” OR “Overall Survival” OR “OS” OR “Disease free survival” OR "Disease-Free Survival" OR “DFS” OR “Progression free survival” OR "Progression-Free Survival" OR “PFS” OR "Prognosis"[Mesh] OR Prognosis OR Prognostic

RESULTS

**PUBMED**

“Mouth Neoplasms” [Mesh] OR “Mouth Neoplasm” OR OSCC OR “Oral Squamous Cell Carcinoma” OR “Oral Squamous Cell Carcinomas” OR “Squamous cell Carcinoma of the Tongue” OR “Squamous cell Carcinomas of the Tongue” OR “Oral Cancer” OR “Oral Cancers”

AND

"Tertiary Lymphoid Structures"[Mesh] OR “Tertiary Lymphoid Structure” OR “Tertiary Lymphoid Structures” OR “Tertiary Lymphoid Organ” OR “Tertiary Lymphoid Organs” OR “Ectopic Lymphoid Follicle” OR “Ectopic Lymphoid Follicles” OR “Ectopic Lymphoid Organ” OR “Ectopic Lymphoid Organs” OR “Intratumoral Lymphoid Structure” OR “Intratumoral Lymphoid Structures” OR “Intratumoral Lymphoid Organ” OR “Intratumoral Lymphoid Organs” OR “Intratumoral Lymphoid Follicles” OR “Intratumoral Lymphoid Follicle” OR “Intra-tumoral Lymphoid Structure” OR “Intra-tumoral Lymphoid Structures” OR “Intra-tumoral Lymphoid Organ” OR “Intra-tumoral Lymphoid Organs” OR “Intra-tumoral Lymphoid Follicles” OR “Intra-tumoral Lymphoid Follicle” OR “B-Cells Follicle” OR “B-cells Follicles” OR “B-Cells Cluster” OR “B-Cell Cluster” OR “B-Cell Clusters” OR “B-Cells Clusters” OR “BCells Follicle” OR “Bcells Follicles” OR “BCells Cluster” OR “BCell Cluster” OR “BCell Clusters” OR “BCells Clusters” OR “tumor-associated immune aggregates” OR “tumor associated immune aggregates”

AND

"Survival"[Mesh] OR "Disease-Free Survival"[Mesh] OR "Survival Analysis"[Mesh] OR "Survival Rate"[Mesh] OR "Progression-Free Survival"[Mesh] OR “Survival” OR “Survival Analysis” OR “Survival Rate” OR “Overall Survival” OR “OS” OR “Disease free survival” OR "Disease-Free Survival" OR “DFS” OR “Progression free survival” OR "Progression-Free Survival" OR “PFS” OR "Prognosis"[Mesh] OR Prognosis OR Prognostic

**SCOPUS**

Mouth Neoplasm OR OSCC OR Oral Squamous Cell Carcinoma OR Oral Squamous Cell Carcinomas OR Squamous cell Carcinoma of the Tongue OR Squamous cell Carcinomas of the Tongue OR Oral Cancer OR Oral Cancers

AND

Tertiary Lymphoid Structure OR Tertiary Lymphoid Structures OR Tertiary Lymphoid Organ OR Tertiary Lymphoid Organs OR Ectopic Lymphoid Follicle OR Ectopic Lymphoid Follicles OR Ectopic Lymphoid Organ OR Ectopic Lymphoid Organs OR Intratumoral Lymphoid Structure OR Intratumoral Lymphoid Structures OR Intratumoral Lymphoid Organ OR Intratumoral Lymphoid Organs OR Intratumoral Lymphoid Follicles OR Intratumoral Lymphoid Follicle OR Intra-tumoral Lymphoid Structure OR Intra-tumoral Lymphoid Structures OR Intra-tumoral Lymphoid Organ OR Intra-tumoral Lymphoid Organs OR Intra-tumoral Lymphoid Follicles OR Intra-tumoral Lymphoid Follicle OR B-Cells Follicle OR B-cells Follicles OR B-Cells Cluster OR B-Cell Cluster OR B-Cell Clusters OR B-Cells Clusters OR BCells Follicle OR Bcells Follicles OR BCells Cluster OR BCell Cluster OR BCell Clusters OR BCells Clusters OR tumor-associated immune aggregates OR tumor associated immune aggregates

AND

Survival OR Overall Survival OR OS OR Disease free survival OR DFS OR Progression free survival OR PFS OR Prognosis OR Prognostic

**EMBASE**

“Mouth Neoplasm” OR OSCC OR “Oral Squamous Cell Carcinoma” OR “Oral Squamous Cell Carcinomas” OR “Squamous cell Carcinoma of the Tongue” OR “Squamous cell Carcinomas of the Tongue” OR “Oral Cancer” OR “Oral Cancers”

AND

“Tertiary Lymphoid Structure” OR “Tertiary Lymphoid Structures” OR “Tertiary Lymphoid Organ” OR “Tertiary Lymphoid Organs” OR “Ectopic Lymphoid Follicle” OR “Ectopic Lymphoid Follicles” OR “Ectopic Lymphoid Organ” OR “Ectopic Lymphoid Organs” OR “Intratumoral Lymphoid Structure” OR “Intratumoral Lymphoid Structures” OR “Intratumoral Lymphoid Organ” OR “Intratumoral Lymphoid Organs” OR “Intratumoral Lymphoid Follicles” OR “Intratumoral Lymphoid Follicle” OR “B-Cells Follicle” OR “B-cells Follicles” OR “B-Cells Cluster” OR “B-Cell Cluster” OR “tumor-associated immune aggregates” OR “tumor associated immune aggregates”

AND

Survival OR “Overall Survival” OR “OS” OR “Disease free survival” OR “DFS” OR “Progression free survival” OR “PFS” OR Prognosis OR Prognostic

**WEB OF SCIENCE**

“Mouth Neoplasm” OR OSCC OR “Oral Squamous Cell Carcinoma” OR “Oral Squamous Cell Carcinomas” OR “Squamous cell Carcinoma of the Tongue” OR “Squamous cell Carcinomas of the Tongue” OR “Oral Cancer” OR “Oral Cancers”

AND

“Tertiary Lymphoid Structure” OR “Tertiary Lymphoid Structures” OR “Tertiary Lymphoid Organ” OR “Tertiary Lymphoid Organs” OR “Ectopic Lymphoid Follicle” OR “Ectopic Lymphoid Follicles” OR “Ectopic Lymphoid Organ” OR “Ectopic Lymphoid Organs” OR “Intratumoral Lymphoid Structure” OR “Intratumoral Lymphoid Structures” OR “Intratumoral Lymphoid Organ” OR “Intratumoral Lymphoid Organs” OR “Intratumoral Lymphoid Follicles” OR “Intratumoral Lymphoid Follicle” OR “B-Cells Follicle” OR “B-cells Follicles” OR “B-Cells Cluster” OR “B-Cell Cluster” OR “tumor-associated immune aggregates” OR “tumor associated immune aggregates”

AND

Survival OR “Overall Survival” OR “OS” OR “Disease free survival” OR “DFS” OR “Progression free survival” OR “PFS” OR Prognosis OR Prognostic

**PUBMED: 18**

**SCOPUS: 18**

**EMBASE: 10**

**WEB OF SCIENCE: 9**
